# Visible-Pump Terahertz Probe Measurements of Embedded Polymer Conductivity in Organic Matrices

**DOI:** 10.3390/polym17233169

**Published:** 2025-11-28

**Authors:** Clyde Varner, Edwin Heilweil

**Affiliations:** 1 Department of Physics, Chemistry, and Mathematics, College of Engineering, Technology and Physical Sciences, Alabama A&M University, Huntsville, AL 35811, USA; 2 Physical Measurement Laboratory, Associate, Nanoscale Device Characterization Division, National Institute of Standards and Technology, Gaithersburg, MD 20899, USA; edwin.heilweil@nist.gov

**Keywords:** polymer, semiconductor, terahertz

## Abstract

We report measurements of ultrafast photoinduced charge separation and recombination processes in the conjugated donor–acceptor (D-A) polymer PSBTBT, both as pure film and blended in various polymer matrices. Using time-resolved terahertz spectroscopy (TRTS), time-dependent photoconductivity is measured for samples with PSBTBT weight fractions (*W_PSBTBT_:W*_PE/PEG/PS_) of 2.0% dispersed in high-density polyethylene (HDPE), polyethylene glycol (PEG), and polystyrene (PS). Charge carrier generation is an intrinsic feature of conductive polymers that occurs on sub-picosecond and longer timescales and is attributed to initially generated dissociation of bound polaron pairs into free carriers that reside on polymer chains, or to adjacent interchain charge transfer and migration. Both interchain and interfacial charge transfer contribute to the measured photoconductivity of the samples, which is found to increase as a function of increasing local polarity and an increasingly hydrogen-bonded environment. Pure-PSBTBT polymer film, PSBTBT dispersed in PS, and PSBTST dispersed in HDPE were all found to exhibit shorter photoconductive free-carrier long-time signal decay than PSBTBT in a hydrogen-bonded, semi-crystalline PEG environment.

## 1. Introduction

Highly stretchable, transparent electronics have become a leading area of research in the advancement of materials [1]. Semiconducting polymers within insulating matrices which express sensitivity as a function of charge carrier (n- or p-type) concentration are now an essential part of wearable electronics [2], solar cells [3], sensors [4], and transistors [1]. Recently, research has also been carried out on developing polymer-based intrinsically stretchable semiconductors. Conjugated polymers (CPs) such as poly (3-hexylthiophene) (P3HT) or PEDOT: PSS are representative polymeric semiconductors that are stretchable and translucent [1,5,6,7,8]. Chortos et al. reported on a P3HT-based semiconductor which had an optical density of 0.26 and a maximum stretchability of 250% [1,8]. Improvement in overall semiconducting properties such as carrier mobility and bandgap is also a key factor in these types of materials; this has been achieved by preparing polymers with conjugated pi-structures, including alternating donor and acceptor moieties [9]. A reduction in matrix crystallinity increases the deformability of conjugated polymers but comes at the price of a reduction in charge carrier mobility [10].

Nanoconfinement is a concept newly introduced to address crystallinity in charge carrier mobility reduction [7]. Xu et al. were able to input conjugated polymeric nanofibrils inside an elastomer matrix [1,7]. The resultant polymer-based semiconductor had a maximum mobility of 1.32 cm^2^V^−1^s^−1^ and an average mobility of 1.08 cm^2^V^−1^s^−1^ at 100% tensile strain, with an on/off ratio of magnitude 105 and current leakage of 10^−9^ A [1,7].

The THz region of the electromagnetic spectrum (~0.1–10 THz) bridges the microwave and mid-infrared regions, and encodes information pertaining to molecular rotations, intermolecular interactions, and phonon activity [11]. This region of the electromagnetic spectrum has become more accessible through the use of ultrafast lasers for THz pulse generation. THz radiation has many applications; these include, but are not limited to security and high-resolution imaging [12], in situ monitoring of cellular systems within biological systems [13], and, as used in this investigation, providing invaluable non-contact measurements of electrical photoconductivity [14,15,16]. Light-induced changes in low-frequency THz sample transmission due to electronic interactions in the semiconductor medium [17] reflect alterations to the conductivity and mobile charge carrier dynamics of the material. Thus, the properties of environmental stress/strain time-dependent electrical conductivity in a highly stretchable, transparent nanoconfined semiconductor in polymer matrix can be determined by examining its photoconductivity through THz spectroscopy. Time-resolved THz spectroscopy (TRTS) is the primary technique for assessing material photoconductivity following optical excitation [18]. Ultrafast TRTS enables polymeric conjugated donor–acceptor-based semiconductors to be measured on picosecond-to-microsecond timescales relevant to various photophysical processes (e.g., energy transfer, charge-separated states, and exciton migration), without the use of electrodes.

Furthermore, the role of solvents in photo-electrochemistry has been extensively explored, with PPY, PANI, polycarbazole, polythiophene, and their derivatives all having been studied for application in biosensors and organic electrochemical transistors (OECTs) (see [19] and references therein). Several groups have shown the versatility of CPs and achieved significant progress in advancing organic electronics. Measurements obtained using time-resolved terahertz spectroscopy of the conjugated D-A polymer PSBTBT (see structure below), consisting of alternating cyclopenta-dithiophene (D-donor) and thiadiazolo-pyridine (A-acceptor) units and related D-A species, were previously examined by our group [19]. While terahertz (THz) conduction in neat-polymeric films is typically evaluated using unspecified polarizations, measurement with specifically oriented pump-probe polarized fields enables extraction of sample-dependent anisotropic conduction. Films prepared using techniques that affect sample morphology, including substrate nanoscale etched grooves and blading speed, and facilitate polymer alignment were compared to more randomized films and liquid toluene dispersions in [19,20,21]. Comparisons were also made among PCDTPT films and dispersions and with the traditional photoconductive polymer poly-3-hexylthiophene (P3HT) in [19]. We also previously reported polarization anisotropy for aligned PCDTPT samples using transient polarized electronic absorption spectroscopy in [19].

## 2. Methods

In this work, the conductivity of PSBTBT dispersed in several visible and THz-transparent polymer matrices is directly measured using linearly co-polarized femtosecond excitation pulses (400 nm and 800 nm) and polarized THz probe pulses. Room-temperature time-resolved terahertz (TRTS) spectroscopic measurements directly yield information about excitation-probe absorption-dependent time-decay dynamics. We previously reported that related donor–acceptor polymers in more isotropic environments (e.g., drop-cast films on nanostructured substrates and toluene dispersions) yield conduction signals comparable to those of ordered films, but that this is not the case for P3HT and other D-A polymers solubilized in chlorinated solvents [14,19]. In the latter work, when chlorinated dispersions were studied, charge transfer to the solvent appeared to occur at a much higher rate than intra- or interchain transfer, and no TRTS signals were observed [19].

Ultraviolet-to-visible absorbance measurements were made using a PerkinElmer Lambda 2 spectrometer NIST Gaithersburg, MD, USA [20] with dual-beam setup for referenced transmission acquisition. * Spectroscopic absorption measurements spanning 1 to 20 THz (33 cm^−1^ to 700 cm^−1^) were made using a modified Nicolet Magna 550 * Fourier-transform infrared spectrometer (FTIR-THz) [22]. Spectra for the four investigated films are displayed in SI Figure S1.

The neat and dispersed PSBTBT film samples were prepared from CP powder as received from Ossilla LTD, Windsor Street, Sheffield, UK * by adding approximately 2.0 weight % PSBTBT (molecular weight in g/mol MW = 95,000 (32)), to 100-micron-particle high-density polyethylene (HDPE, Aldrich MW~135,000), polystyrene (PS, Aldrich MW = 350,000), or polyethylene glycol flakes (PEG, Aldrich MW = 2050). The polymer mixtures were uniformly and fully dispersed at room temperature using suitable solvents: dichloromethane (DCM) for most polymers, and toluene for HDPE-based films. For polymers with limited solubility (e.g., HDPE), an emulsion technique was employed to enhance dispersion. The polymers were first dispersed in toluene with agitation to form a stable emulsion, ensuring uniformity before drop-casting onto THz-transparent amorphous silica windows. This process minimized aggregation and improved film homogeneity, particularly in HDPE films. During film preparation, there was some exposure to atmospheric moisture; this was subsequently removed by purging the films in a dry FTIR sample chamber or evacuating them in a glove box. After deposition, HDPE films were further annealed at 420 K for an hour to remove any residual toluene or trapped gases, ensuring homogeneity and uniformity.

Contact atomic force microscopy (Nanosurf Easyscan 2 *) Nanosurf AG Liestal Switzerland was used to map the surface topography for possible inhomogeneous structures in the deposited polymer films. Potential PSBTBT structural alignment was investigated using these measurements. The resulting 10 × 10 micron AFM contact topography images for the three polymer matrices may be found in Figure S2 of the SI. A home-built cross-polarized imager using two sheet polarizers (Thorlabs Inc., Newton, NJ, USA) and magnifying lens was employed to analyze any film inhomogeneity and any related degree of crystallization (via polarization rotation) [23] leading to local structural changes that might affect the dispersed PSBTBT conductivity (see SI Figure S3).

**Figure 1:**
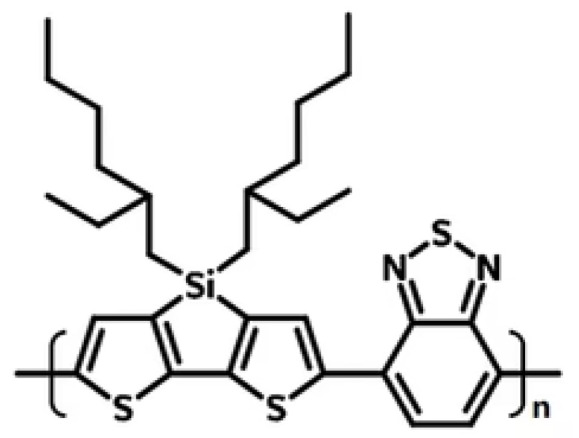
Structure of PSBTBT.

As shown in Figure 1 (left), the TRTS measurement apparatus is built around an amplified femtosecond Ti:sapphire laser system (Coherent MIRA Seed and Legend regenerative amplifier Coherent soxonburg Pennsylvania) [21,24,25] operating at a 1 KHz repetition rate, as described in detail previously [14]. Certain commercial equipment, instruments, or materials are identified in this paper to foster understanding. Such identification does not imply recommendation or endorsement by the National Institute of Standards and Technology, nor does it imply that the materials or equipment identified are necessarily the best available for the purpose. In summary, amplified 800 nm pulses (ca. 1.6 mJ/pulse, 40 fs FWHM duration) are split into three delay arms for generation of visible excitation/pump pulses, generation of the THz probe pulses, and gated electro-optic detection of the THz probe pulses transmitted through the sample. THz probe pulses are generated by optical rectification of 800 nm pulses using a 1 mm thick ZnTe<110> crystal and electrooptically sampled by weak 800 nm gate pulses using a 2 mm thick ZnTe<110> detector crystal [26]. Electric field information contained within the transmitted THz probe pulse is carried by the 800 nm gate pulses which are polarization-selected and collected by a pair of balanced silicon photodetectors using lock-in amplification (Stanford Research Systems, SR830, Sunnyvale, CA, USA). THz generation and detection are performed inside a closed sample chamber purged with dried air to avoid absorption of THz probe and sample by atmospheric water.

**Figure 2:**
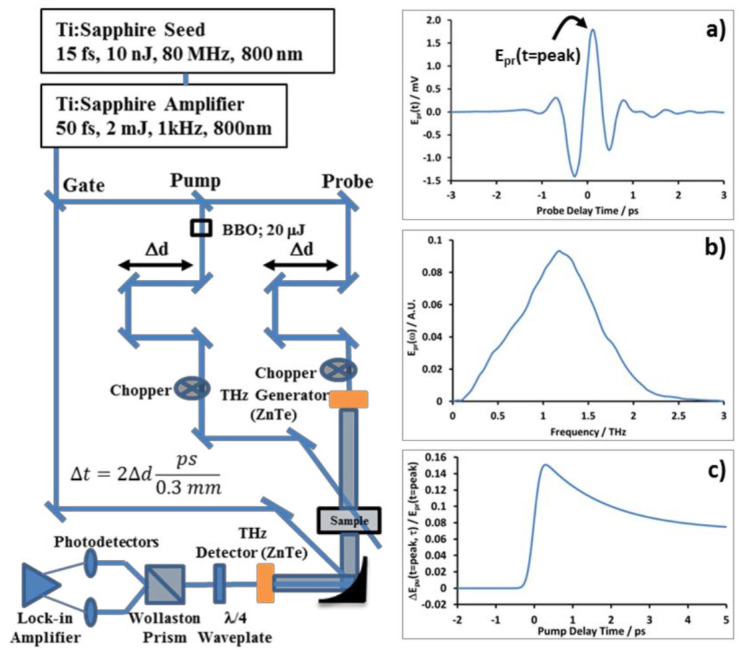
Left: Schematic diagram of the TRTS apparatus. (**a**) A typical THz waveform, as measured by our spectrometer. (**b**) The Fourier transform of a typical THz waveform. (**c**) A representative pump scan showing the dynamics of photoconductivity.

Electro-optic detection allows for measurement of the amplitude and phase of the THz probe pulse electric field, E_pr_(t). By scanning the probe delay time, t, relative to the gate pulse while modulating the probe, E_pr_(t) is obtained, as shown in Figure 2a. E_pr_(t) can then be Fourier-transformed to E_pr_(ω) (see Figure 2b) and used to determine the absorption coefficient, index of refraction, and complex-valued photoconductivity of a sample relative to a reference sample in the 0.5–2.5 THz frequency range [17]. When applied in this way, the technique is known as terahertz time-domain spectroscopy, THz-TDS [21,24,25,27]. Alternatively, changes in the THz electric field induced in the sample by a visible pump pulse, ΔE_pu_(t,τ), can be measured in two different ways by modulating the pump pulse. When the probe delay is set to the peak of the THz waveform and the pump delay scanned (e.g., time-resolved terahertz spectroscopy or TRTS), the dynamics of the pump-induced absorption changes, ΔE_pu_(t = peak,τ), averaged over all THz frequencies, can be collected, as shown in Figure 2c.

## 3. Results

**Figure 3:**
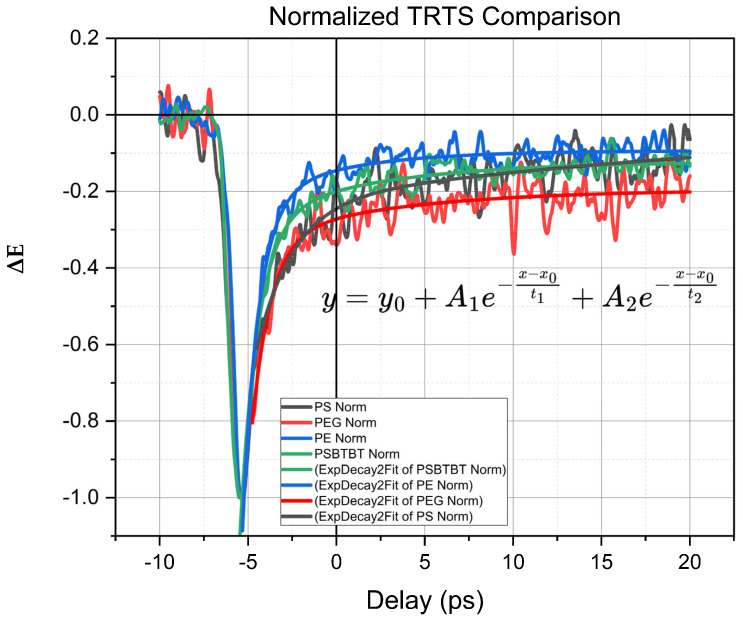
Overlayed normalization of the four TRTS decays (see SI Figure S7) for comparison of both the peak and tail photoconductive signals from drop-cast PSBTBT dispersion films and pure-PSBTBT samples. Photoexcitation was performed using 800 nm pulses.

Donor–acceptor conducting polymers initially conduct polaron charge primarily through intramolecular channels, and they exhibit extensive intramolecular delocalization similar to that exhibited by other donor–acceptor copolymers [24] However, the dependence of charge conduction on polymer sample local morphology is not well understood. To address this lack of information, we employed TRTS conductivity measurements of PSBTBT in neat film and dispersed in the three polymer matrix thin films with different chemical structures. Figure 3 shows the overlapping “peak” normalized TRTS responses for the four film samples; in the figure, a contrast in the matrix-dependent conductivity may be observed in both the initially generated polaron and the longer-delay time non-zero signal “tail” from photoconductive free-carriers. By normalizing the time-dependent TRTS scans to their “peak” maximum signal amplitude, variations in sample optical density (OD) fluctuations in pump pulse power are removed, along with reflective losses from the films. Thus, the relative change in TRTS “tail” signal amplitudes (e.g., y_o_ from fitting) arises from sample-induced charge localization [25], trapping of charges [28], and a decreased probability of charge transfer upon each scattering event, compared to the initially generated hot polarons. The TRTS “tail” signal magnitude indicates that following initial charge recombination, energy dissipation processes, and trapping (all of which occur within the first few picoseconds), the conductive polarons behave similarly, regardless of whether they reside on electronically uncoupled or isolated strands immersed in a dielectric polymer matrix, compared with the two-dimensional conductive network coupled to the surrounding polymer. Values of absolute conductivity and mobility were difficult to compare, given the differences in sample densities and the heterogeneous nature of the polymer films. However, the relatively long time period of “free” carrier behavior is comparable in all polymer matrix environments in the thermally relaxed (>few ps) limit.

**Figure 4:**
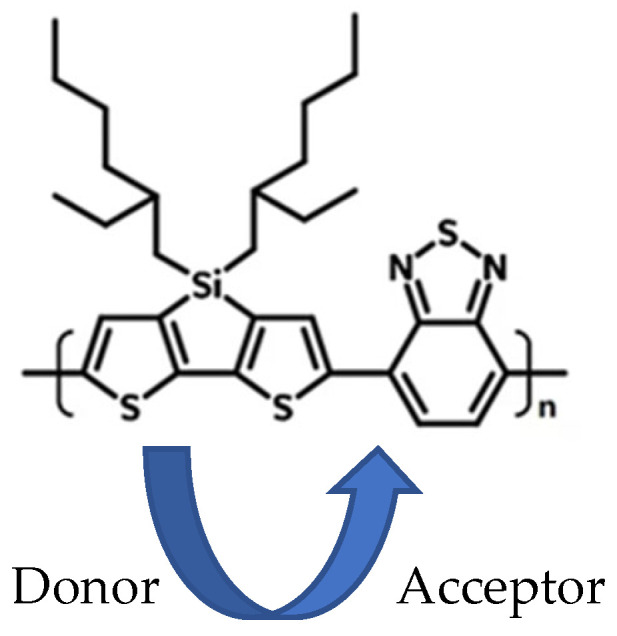
Label of donor and acceptor portions of PSBTBT.

Upon photoexcitation, intermolecular orientation, polycrystalline domains, and stabilization of the nanoscale film structure all may affect intrachain charge motion in the pure-PSBTBT film Figure 4, compared to the conducting polymer dispersed within other polymer matrices. On the 20 ps timescale, free-carrier “tail” dynamics and signal levels (see TRTS scans with biexponential fits provided in Figure 2, and y_o_ offsets provided in SI Tables S1–S4) manifest in different scattering event length scales and a greater proclivity for motion along a given strand. We find that the pure-PSBTBT film exhibits an intermediary “tail” signal level (see the y_o_ fitting parameters) when compared to the highly polar hydrogen-bonded PEG environment. On the other hand, the PS matrix appears to inhibit long-lived charge conductivity, perhaps due to a more inhomogeneous or lower-crystallinity environment for the conductive polymer. The PEG matrix long-time “tail” conductivity level is enhanced by a factor of 1.55 compared to that for PSBTBT in PS.

To further investigate film characteristics potentially responsible for the observed changes in long-lived conductivity, we performed contact AFM surface topography/phase measurements on all four films. Shown in SI Figures S2–S5 are the resultant 10 × 10 micron images for each film, taken at two different surface positions for comparison purposes. The pure-PSBTBT film surface (Figure S2) is relatively smooth, with no indication of any of the several “dip” features which may arise from escaping gas during deposition. The HDPE film (Figure S3) also appears smooth, with very little surface sub-structure. The PS film (Figure S5) yielded larger globular structures. Contrarily, the PEG film (see Figure S4 phase images) produced localized linear structural regimes which may have arisen from semi-crystalline domains.

In order to assess more critically the structural properties of the films, we investigated potential visible-light depolarization effects which could arise from amorphous or crystalline domains [23]. The acquired images obtained through crossed-polarizers are shown in SI Figure S3. These images help identify high crystallinity (strong depolarization; white-light transmission) compared to more amorphous film structures (weak depolarization; near-black regions) in the matrix films. The pure-PSBTBT film exhibits weak depolarization (MW = 95,000; predominantly amorphous) while the pure-PS film is strongly depolarized overall, with a few very localized spots (MW = 350,000; yielding isolated Maltese crosses). The two PSBTBT-doped HDPE films show the lowest level of depolarization, suggesting they are nearly amorphous with few or no crystalline domains (PE MW = ~135,000). Conversely, the pure-PEG film (with lowest MW = 2050) and the PSBTBT-doped films produced very strong depolarization images, with dendrites suggesting that PEG may help orient the dilute PSBTBT along matrix domains.

**Figure 5:**
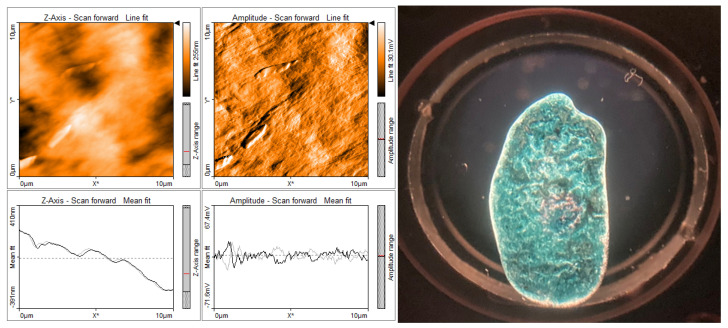
Comparison of (**Left**) AFM images of PSBTBT 2 wt% in polyethylene glycol (PEG) with arbitrary film orientation showing striated sub-micron features in both topology and phase images and (**Right**) cross-polarization image of PSBTBT in PEG matrix (2.5 cm edge-to-edge).

We suggest that because the THz probe beam samples millimeter-sized regions of the films, the AFM and polarization images provide evidence for why each matrix yields different long-time conductivity. The neat-PSBTBT film and the PS matrix yielded nearly identical TRTS responses and yielded fairly flat, unstructured AFM and low-depolarization images. These results suggest that similar local amorphous structures exist in these specific films. The PSBTBT-doped PE film, which produced a predominantly flat AFM surface and yielded no depolarization (e.g., isotropic) is likely the most amorphous, permitting the PSBTBT to be coiled or self-aggregated and thus produce the lowest conductivity response. Most striking are the pure- and doped-PEG films—the AFM images show strong striations on the micron scale while the polarization images exhibit very strong depolarization (Figure 5, Left). These dendrites are also clear in the cross-polarized white-light images in Figure 5, Right and SI Figure S6.

Table 1 includes values reported in the literature which indicate that PEG exhibits extremely high crystallinity and has the lowest melting point, in comparison with the other polymers. We believe the micron-scale PEG molecules act as internal templates for the PSBTBT to extend along, thus providing a more extended structure that increases local conductivity and hence produces the largest “tail” TRTS response. In [25], similar behavior was reported for donor–acceptor polymers aligned along nano-grooved substrates. PSBTBT possesses physical and electronic properties beyond its nominal number-average molecular weight that directly influence its charge transport behavior in both neat films and dispersed matrices. The polymer features alternating cyclopenta-dithiophene donor and thiadiazolo-pyridine acceptor units; these yield a low optical bandgap (~1.6 eV), a stabilized intramolecular charge-transfer excited state, and delocalized hole polarons following photoexcitation. The backbone exhibits low torsional disorder and a persistence length of the order of several nanometers, enabling intrachain conjugation lengths spanning ~15–25 repeat units in well-packed domains. These structural and electronic features distinguish PSBTBT from more flexible or torsionally labile conjugated polymers such as MEH-PPV, which show much greater exciton localization and exhibit a correspondingly lower photoconductive response when dispersed in matrices. The molecular weight (MW) plays a pivotal role in determining whether the conjugated chains are long enough to bridge microdomains and support continuous polaron transport pathways. In the case of PSBTBT, the MW used here (~95 kDa) lies near the range where chains are sufficiently extended to maintain coherent delocalization, while still avoiding excessive entanglement that would disrupt π-stacking. Additionally, the donor and acceptor monomeric units differ in size and steric influence, with the bulkier donor segment enforcing backbone planarity and the smaller acceptor stabilizing charge-transfer states, producing a chain architecture whose electronic delocalization is highly sensitive to conformational constraint. As a result, matrix-induced confinement becomes a critical determinant of long-time TRTS conductivity: amorphous matrices such as PS and HDPE permit chain coiling and reduced conjugation length, whereas the semi-crystalline PEG environment templates backbone extension and the π-stacking registry, thereby increasing polaron coherence length and long-time free-carrier conductivity. Thus, the observed matrix-dependent differences in TRTS tail amplitudes originate from the interplay of intrinsic PSBTBT electronic structure, chain length, and matrix-imposed conformational confinement controlling the delocalization of photoinduced charge carriers.

## 4. Conclusions

Thermal dissipation may play a role in liquid and matrix dispersions because a dispersed sample absorbs a significantly smaller volume of pump light than pure film, leading to a much greater potential for local heating. However, it is unlikely that thermal effects would be dominant on a 20 ps timescale because thermal transport dynamics in liquid and solid samples generally occur on timescales of nanoseconds or longer. Follow-up studies could be conducted at lower fluences and different temperatures to isolate this potential effect.

The finding that dispersion of a donor–acceptor conjugated polymer (e.g., PSBTBT) in organic polymer matrices yields TRTS signals comparable to those of the analogous pure-PSBTBT film is significant. Other polymers, such as MEH-PPV, do show isolated, solvated conduction, but with far less conductivity than in films [22], while PSBTBT produces comparably higher conductivity under either circumstance [19]. The effect of confinement of P3HT in PMMA films from the photophysical environment has also been studied [22]. Lastly, the observation that dispersed PSBTBT retains photoconductive amplitude comparable to that of neat-PSBTBT film is significant because it demonstrates that long-range electronic delocalization can persist even when the conjugated backbone is spatially confined within a foreign polymer matrix. This behavior contrasts sharply with that of other conjugated polymers such as MEH-PPV, where solvation or matrix dilution typically yields isolated excitons and highly localized polarons, resulting in greatly reduced photoconductivity relative to the solid film phase at similar pump fluence [22]. By comparison, PSBTBT maintains a larger effective polaron delocalization length and greater interchain electronic coupling, which enables mobile charge carriers to survive beyond the sub-picosecond exciton dissociation regime and contribute to the long-time THz “tail” conductivity [19]. This behavior directly reflects the role of confinement. When a donor–acceptor polymer is dispersed in a matrix, the degrees of chain extension, torsional planarity, and π–π stacking registry determine whether charge carriers propagate along the backbone or become trapped at local disorder sites. In weakly interacting, amorphous matrices such as PS or HDPE, the polymer chains adopt coiled or folded conformations which interrupt through-bond conjugation and suppress interchain resonant charge transfer. This yields shorter polaron coherence lengths, resulting in a lower long-time photoconductive response. In contrast, PEG introduces semicrystalline lamellae that serve as physical alignment scaffolds, guiding the PSBTBT chains into more extended, more planar conformations. This reduces torsional disorder, increases interchain electronic orbital overlap, and enhances polaron delocalization, collectively increasing the free-carrier yield and mobility observed in the TRTS tail region. This matrix-induced templating effect is analogous to nanogroove-assisted chain alignment and to nanoconfinement in PMMA–P3HT composites, where the host matrix dictates the dimensionality and directionality of charge migration pathways [22].

Thus, the ability of PSBTBT to maintain and even enhance long-lived conductivity when confined in a highly ordered PEG environment reveals that the matrix is not a passive diluent, but rather an active structural determinant of electronic transport topology in the composite. This highlights confinement as a design variable for controlling polaron coherence, exciton dissociation, and intramolecular versus intermolecular charge transport in donor–acceptor polymer systems.

Comparison of nominally isotropic and partially crystalline PSBTBT drop-cast films using transient terahertz-TRTS absorption measurements revealed differences in the dynamics of the photoinduced charge carrier populations and their relative conductivities. For PSBTBT dispersed in low-molecular-weight PEG, long-time signal amplitude and relaxation time were found to be greater than for neat and other polymer matrices. The more crystalline PEG matrix appears to serve as a template for the conducting PSBTBT polymer, and it enhances long-time conductivity by approximately a factor of two. This series of analyses provides a methodology for assessing the local and directional efficacy of comparable materials in facilitating charge transport and increasing conductivity, when used as components in organic photovoltaic devices and related applications. While these analyses cannot be used to predict device performance or DC conductivity limits, they may provide insight when identifying the roots of photoactive charge separation and initial/bulk transport that underlie conductivity in photoactive materials, and they could be of paramount importance in material device design. We plan to extend these early studies of related D-A conducting polymeric systems to more quantitatively investigate and contrast their behavior when prepared as films and dispersions with the behavior of PSBTBT embedded in these and other polymeric matrices reported in the present study.

## Figures and Tables

**Table 1:** Polymer melting-point and crystallinity values. See Refs. [28,29,30,31].

Polymer	MW (g/mol)	Melting Point (deg C)	Degree of Crystallinity (%)
HDPE	~135,000	120–140	30% to 50% [31]
PS	350,000	240–260	<45% (syndiotactic) [29,30,31]
PEG	2050	64–66	ca. 86% [28]
PSBTBT	95,000	NA	NA

## Data Availability

The original contributions presented in this study are included in the article/Supplementary Materials. Further inquiries can be directed to the corresponding author.

## Supplementary Materials

The following supporting information can be downloaded at: https://www.mdpi.com/article/10.3390/polym17233169/s1, Figure S1: UV-Vis Absorbance Spectra of PSBTBT Pure Sample and Embedded in HDPE, PEG, and PS; Fitting Parameters derived from TRTS Decay Curves (Table S1: Polystyrene (PS); Table S2: High Density Polyethylene (HDPE); Table S3: Polyethylene Glycol (PEG); Table S4: Neat PSBTBT); AFM Surface Topography Scans of PSBTBT Neat and Dispersed in PS, HDPE and PEG Polymer Matrices (10x10 micron scale) (Figure S2: Neat PSBTBT film at two different locations (topography left, phase right); Figure S3: PSBTBT in Polyethylene (HDPE, 2 wt%) at two locations exhibiting mostly flat, smooth and uniform amorphous surface structure in both topology and phase images; Figure S4: PSBTBT 2 wt% in Polyethylene Glycol (PEG) at two locations with arbitrary film orientation showing striated sub-micron features in both topology and phase images; Figure S5: PSBTBT 2 wt% in Polystyrene (PS) at two locations exhibiting mostly flat, smooth and uniform amorphous surface structure in both topology and phase images); Figure S6 A–G: Shows Crossed polarizer images of matrix polymers and 2 wt% PSBTBT films used in TRTS studies; Figure S7: TDS (0 to 3 THz) and TRTS Absorption Spectrum of 2 wt% PSBTBT in Polystyrene; Figure S8: TDS and TRTS 2 wt% PSBTBT in Polyethylene glycol (PEG); Figure S9: TDS and TRTS 2 wt% PSBTBT in High Density Polyethylene (HDPE); Figure S10: TDS and TRTS of neat PSBTBT film.
