# Supplementary material for: Visible-Pump Terahertz Probe Measurements of Embedded Polymer Conductivity in Organic Matrices
Source: Polymers (Basel). 2025 Nov 28;17(23):3169. doi: 10.3390/polym17233169 (PMC12693946; doi:10.3390/polym17233169)

Supplementary Information:

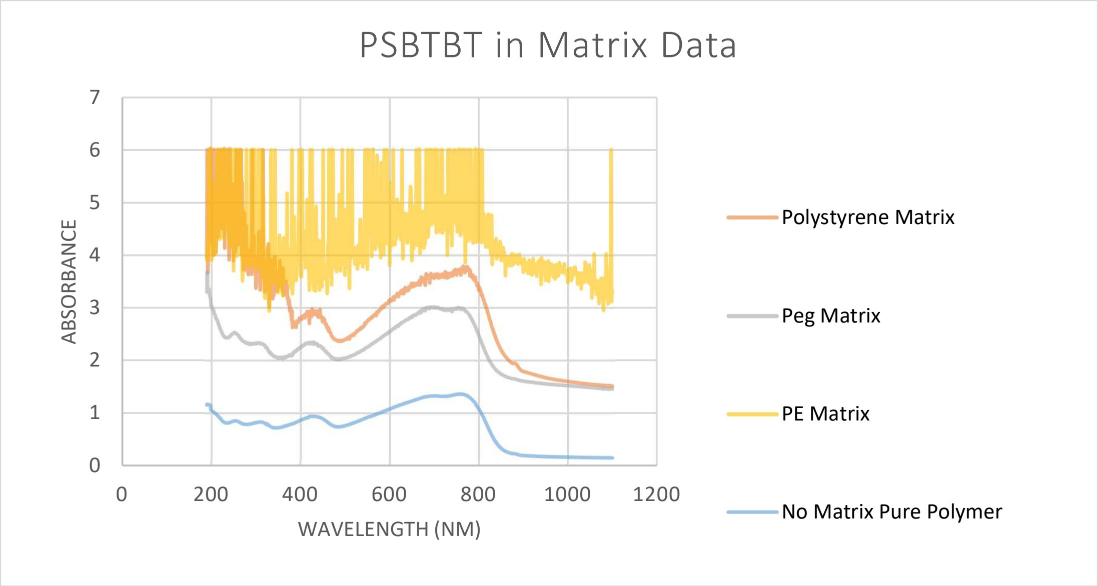

Figure S1. UV-Vis Absorbance Spectra of PSBTBT Pure Sample and Embedded in HDPE, PEG, and PS. Large HDPE powder size (ca. 100μm) gives rise to the off-scale (scatter) absorbance >3.5 for that film measurement

Fitting Parameters derived from TRTS Decay Curves (see text Figure 2):

Table S1. Polystyrene (PS).

Model	ExpDecay2
Equation	y = y0 + A1*exp(-(x-x0)/t1) + A2*exp(-(x-x0)/t2)
Plot	PS Norm
y0	-0.1257 ± 0.00331
x0	-4.50869 ± --
A1	-0.60177 ± --
t1	0.93203 ± 0.0609
A2	-0.34042 ± --
t2	5.40157 ± 0.41998
Reduced Chi-Sqr	0.00165
R-Square (COD)	0.93492
Adj. R-Square	0.93466

Table S2. High Density Polyethylene (HDPE).

Model	ExpDecay2
Equation	y = y0 + A1*exp(-(x-x0)/t1) + A2*exp(-(x-x0)/t2)
Plot	PE Norm
y0	-0.09385 ± 0.0018
x0	-5.25781 ± 344931.15407
A1	-0.79673 ± 307785.99149
t1	0.89288 ± 0.02521
A2	-0.13645 ± 9082.4423
t2	5.18193 ± 0.58009
Reduced Chi-Sqr	6.34039E-4
R-Square (COD)	0.96763
Adj. R-Square	0.9675

Table S3. Polyethylene Glycol (PEG).

Model	ExpDecay2
Equation	y = y0 + A1*exp(-(x-x0)/t1) + A2*exp(-(x-x0)/t2)
Plot	PSBTBT Norm
y0	-0.12225 ± 0.00267
x0	-5.35882 ± --
A1	-0.73795 ± --
t1	1.00268 ± 0.02421
A2	-0.15764 ± --
t2	7.16024 ± 0.67862
Reduced Chi-Sqr	5.45972E-4
R-Square (COD)	0.97391

Table S4. Neat PSBTBT.

Model	ExpDecay2
Equation	y = y0 + A1*exp(-(x-x0)/t1) + A2*exp(-(x-x0)/t2)
Plot	PEG Norm
y0	-0.19528 ± 0.0063
x0	-4.93988 ± --
A1	-0.56946 ± --
t1	1.06828 ± 0.07422
A2	-0.13052 ± --
t2	8.17434 ± 1.91016
Reduced Chi-Sqr	0.00164
R-Square (COD)	0.84296
Adj. R-Square	0.84233

AFM Surface Topography Scans of PSBTBT Neat and Dispersed in PS, HDPE and PEG Polymer Matrices (10x10 micron scale)

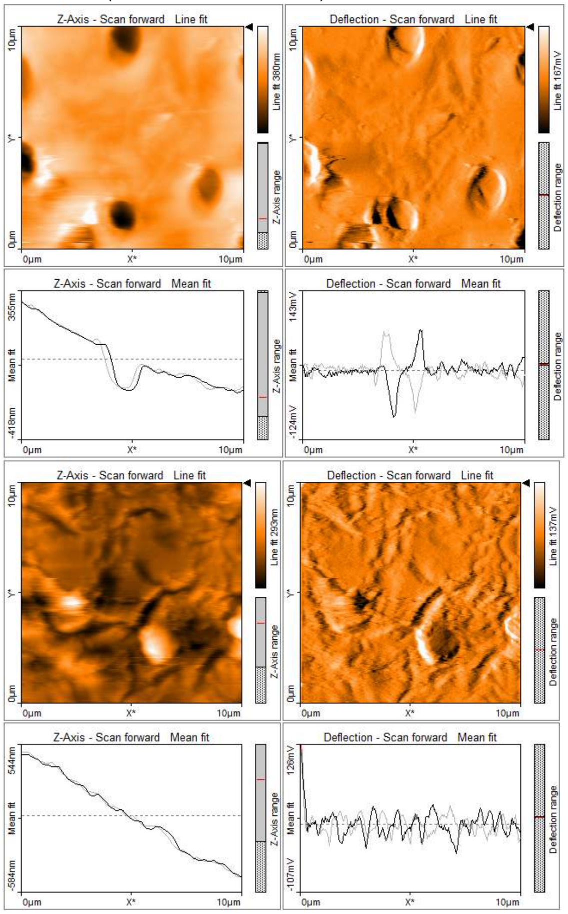

Figure S2. Neat PSBTBT film at two different locations (topography left, phase right). Film appears to have micron-sized voids perhaps from heating and removal of trapped gasses during preparation.

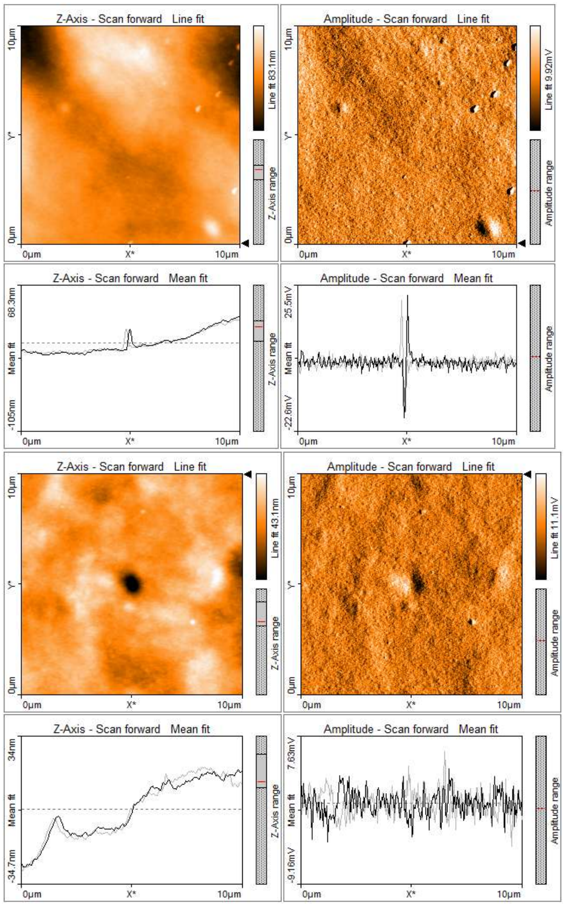

Figure S3. PSBTBT in Polyethylene (HDPE, 2 wt%) at two locations exhibiting mostly flat, smooth and uniform amorphous surface structure in both topology and phase images.

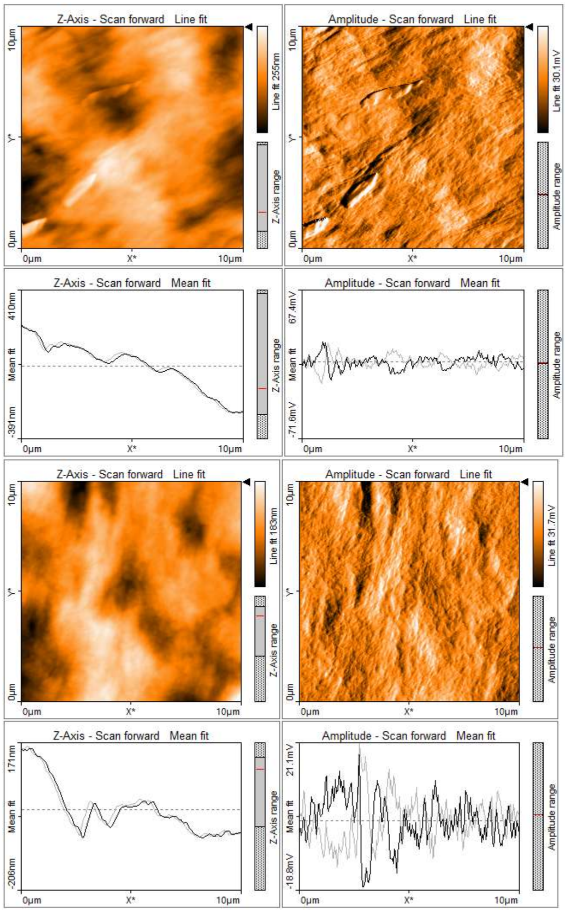

Figure S4. – PSBTBT 2 wt% in Polyethylene Glycol (PEG) at two locations with arbitrary film orientation showing striated sub-micron features in both topology and phase images.

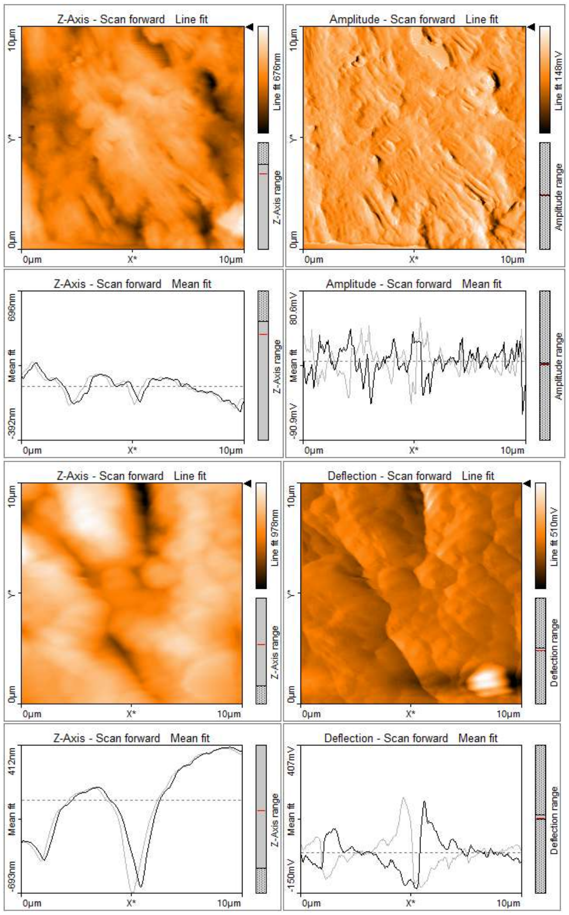

Figure S5. PSBTBT 2 wt% in Polystyrene (PS) at two locations exhibiting mostly flat, smooth and uniform amorphous surface structure in both topology and phase images.

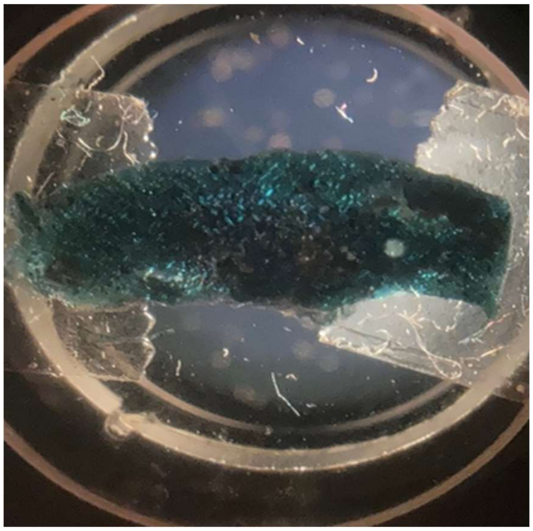

A: PSBTBT in PS film

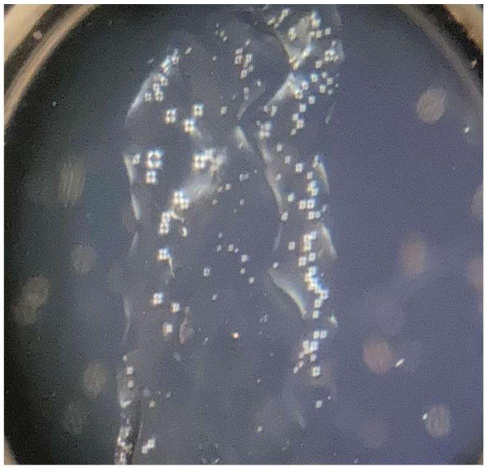

B: Pure PS: (note localized crystallization)

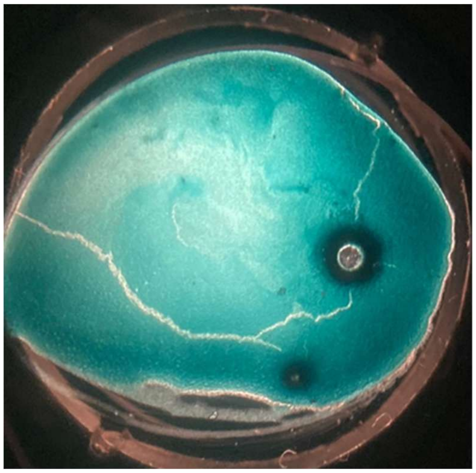

C: PSBTBT in PE for TRTS measurement

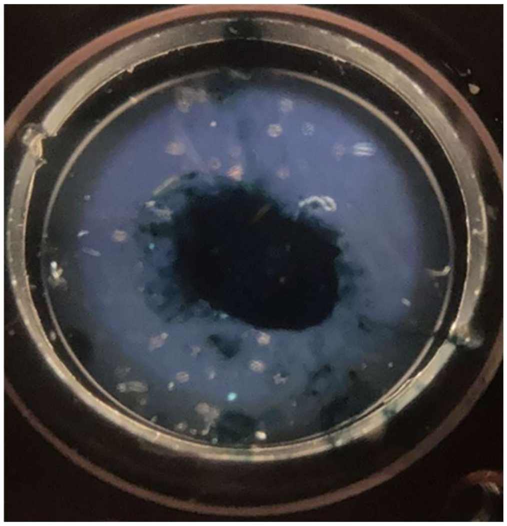

D: PSBTBT Pure Film

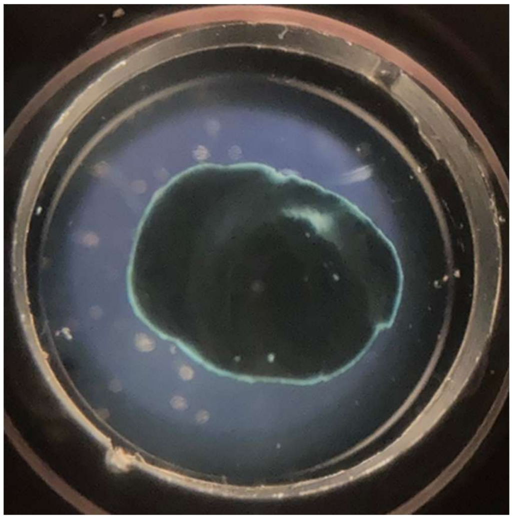

E: PSBTBT in HDPE for TRTS measurement

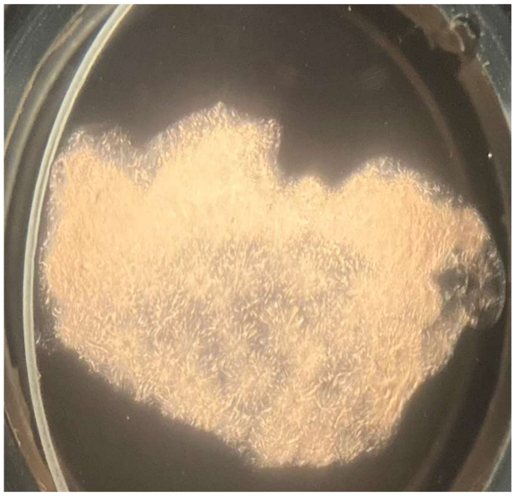

F: Pure PEG clear top photo: (note dendrite-like crystallization).

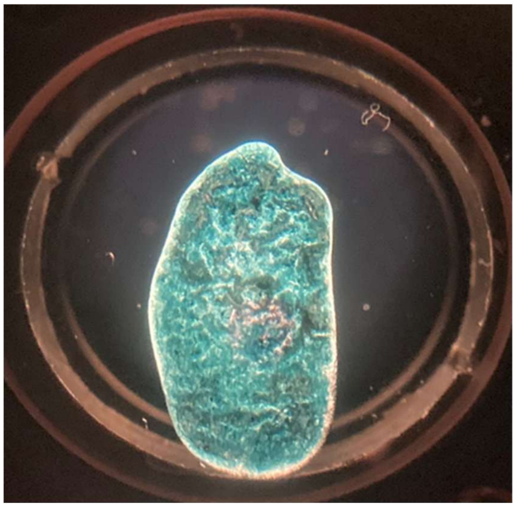

G: Bottom Photo PSBTBT in PEG for TRTS measurement. Cross-polarizer white light images (25 mm edge-to-edge) of pure polymer matrix films and incorporated 2.0 wt% PSBTBT conducting polymer.

Figure S6 A-G: Shows Crossed polarizer images of matrix polymers and 2 wt% PSBTBT films used in TRTS studies. A higher degree of sample crystallinity rotates the polarized light (lighter image) while minimal crystallinity transmits light with no rotation (darker images). Recrystallized low molecular weight (MW) polymers typically show strong crystallization and light rotation using polarized light analysis (e.g., PEG MW=2050 is the lowest) while higher MW species are less likely to crystallize and typically amorphous (e.g., PS at MW=350,000, HDPE MW~135,000 and PSBTBT MW=95,000).*(24)* It is unclear why the 2 wt% PSBTBT in PS image is depolarized relative to the unpolarized neat film (amorphous with local Maltese cross crystallites) image.

THz data for neat PSBTBT and 2 wt% PSBTBT in PS, PEG and HDPE matrices. For each sample type, the THz spectrum (via TDS) for the neat matrix polymer and with 2 wt% PSBTBT are shown. Time-dependent averaged pump-probe (TRTS) scans (pump delay from -5 ps to 20 ps) are also presented.

Fgure S7 TDS (0 to 3 THz) and TRTS Absorption Spectrum of 2 wt% PSBTBT in Polystyrene:

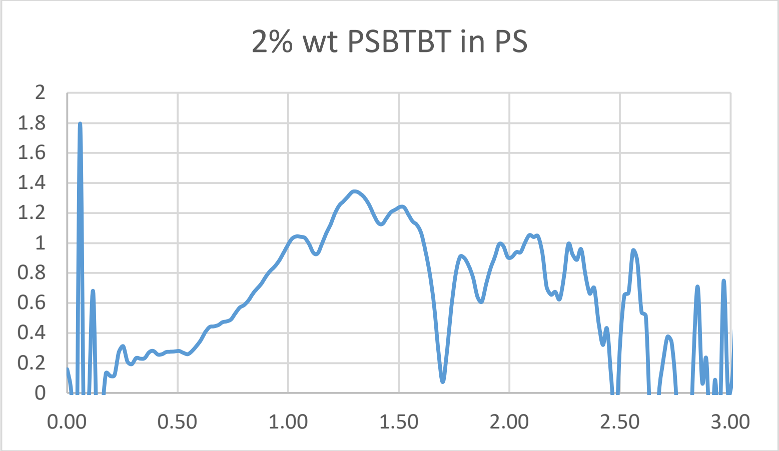

TDS of Pure Polystyrene PS film:

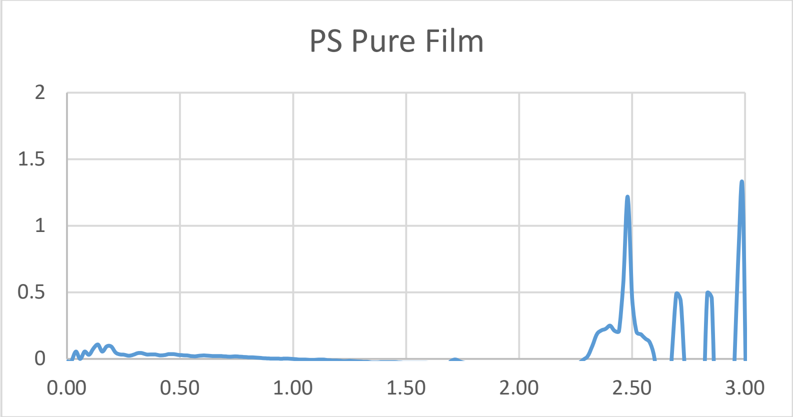

TRTS response (-10 to 20 ps delay) of 2 wt% PSBTBT in Polystyrene

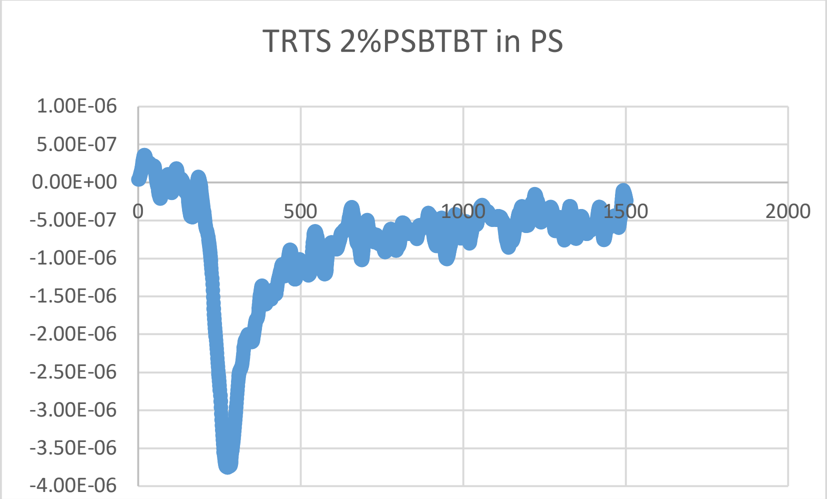

Figure S8: TDS and TRTS 2 wt% PSBTBT in Polyethylene glycol (PEG)

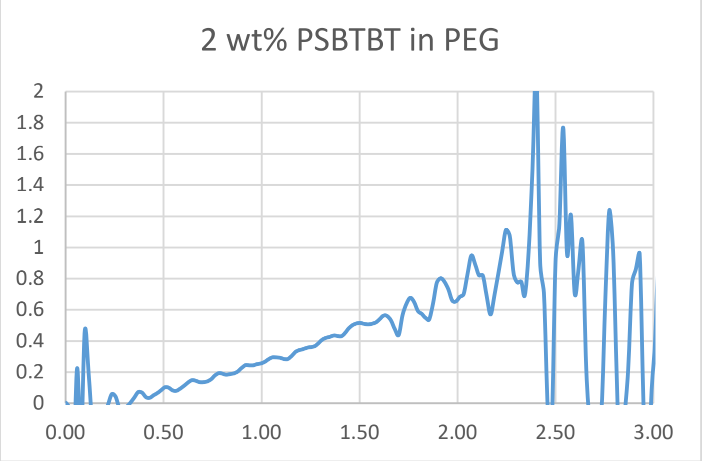

TDS of Polyethylene glycol (PEG)

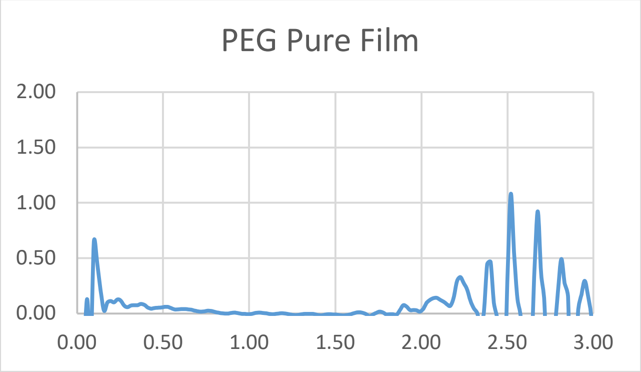

TRTS 2 wt% PSBTBT in Polyethylene glycol (PEG)

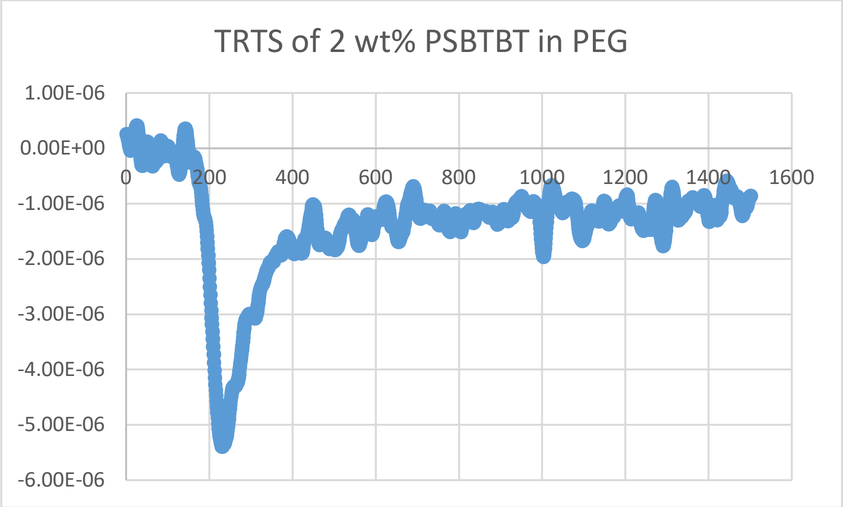

Figure S9: TDS and TRTS 2 wt% PSBTBT in High Density Polyethylene (HDPE)

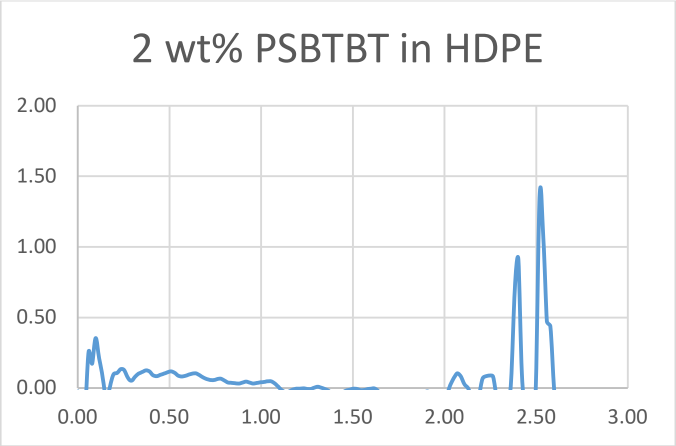

TDS of Polyethylene

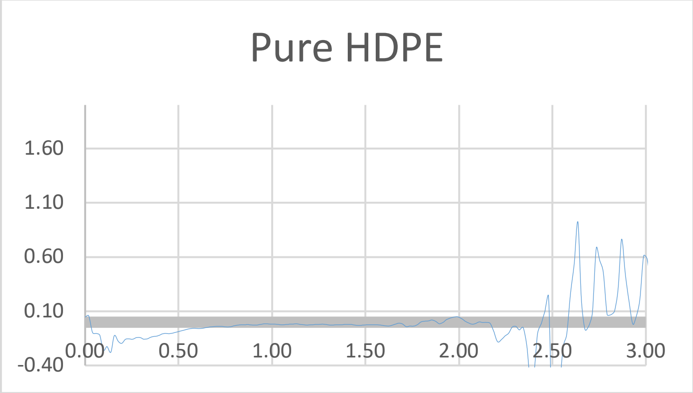

TRTS 2 wt% PSBTBT in Polyethylene (HDPE

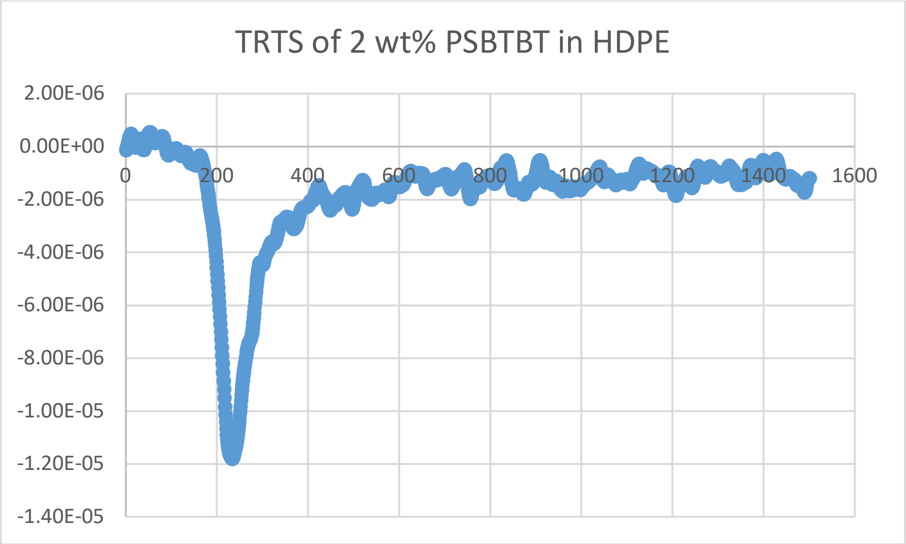

Figure S10: TDS and TRTS of neat PSBTBT film

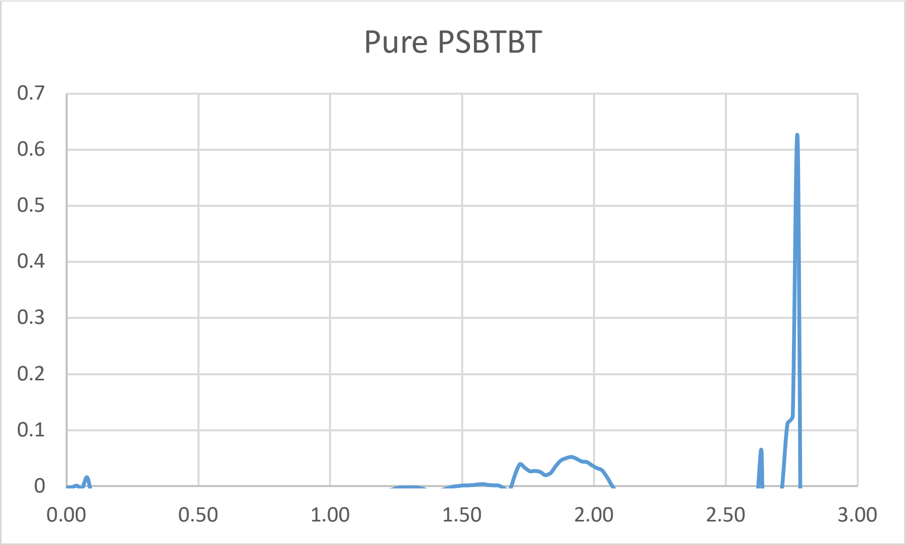

TRTS PSBTBT film

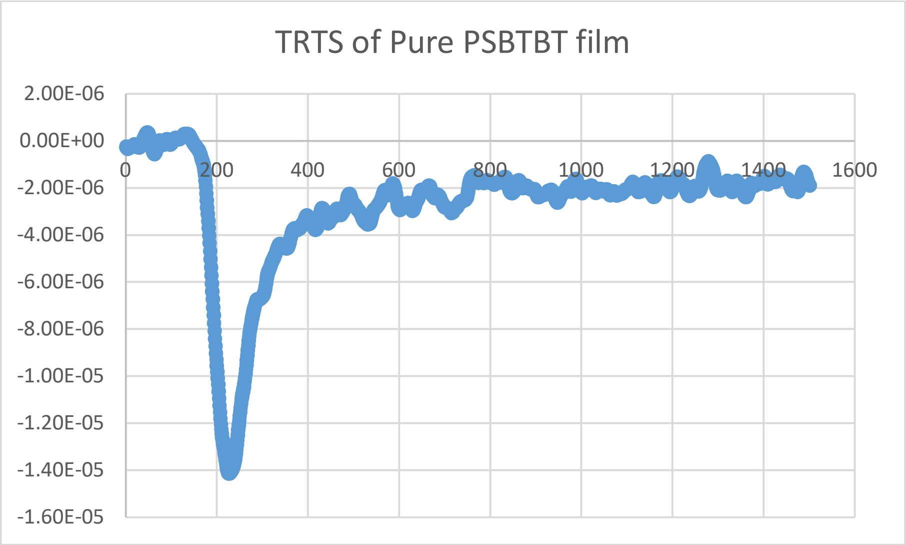

Supplement: Supplementary file 1 [file polymers-17-03169-s001.zip › polymers-3946563-supplementary.pdf]
